# Experiences with tailoring of primary diabetes care in well-organised general practices: a mixed-methods study

**DOI:** 10.1186/s12913-021-07198-2

**Published:** 2021-11-09

**Authors:** Sytske van Bruggen, Marise J. Kasteleyn, Simone P. Rauh, Julia S. Meijer, Karin J. G. Busch, Mattijs E. Numans, Niels H. Chavannes

**Affiliations:** 1 Department of Public Health and Primary Care, Leiden University Medical Center, room V6.26, Postbus 9600 2300, RC Leiden, The Netherlands; 2 Hadoks (Elzha), President Kennedylaan 15, 2517 JK The Hague, The Netherlands; 3 Dept of Epidemiology and Biostatistics, Amsterdam Public Health, Amsterdam UMC, Vrije Universiteit Amsterdam. De Boelelaan 1089a, 1081 HV Amsterdam, The Netherlands; 4 HSK Group, President Kennedylaan 19, 2517 JK The Hague, The Netherlands

**Keywords:** Tailoring of diabetes care, Successful implementation of self-management, Patient experiences

## Abstract

**Background:**

Dutch standard diabetes care is generally protocol-driven. However, considering that general practices wish to tailor diabetes care to individual patients and encourage self-management, particularly in light of current COVID-19 related constraints, protocols and other barriers may hinder implementation. The impact of dispensing with protocol and implementation of self-management interventions on patient monitoring and experiences are not known. This study aims to evaluate tailoring of care by understanding experiences of well-organised practices 1) when dispensing with protocol; 2) determining the key conditions for successful implementation of self-management interventions; and furthermore exploring patients’ experiences regarding dispensing with protocol and self-management interventions.

**Methods:**

in this mixed-methods prospective study, practices (*n* = 49) were invited to participate if they met protocol-related quality targets, and their adult patients with well-controlled type 2 diabetes were invited if they had received protocol-based diabetes care for a minimum of 1 year. For practices, study participation consisted of the opportunity to deliver protocol-free diabetes care, with selection and implementation of self-management interventions. For patients, study participation provided exposure to protocol-free diabetes care and self-management interventions.

Qualitative outcomes (practices: 5 focus groups, 2 individual interviews) included experiences of dispensing with protocol and the implementation process of self-management interventions, operationalised as implementation fidelity. Quantitative outcomes (patients: routine registry data, surveys) consisted of diabetes monitoring completeness, satisfaction, wellbeing and health status at baseline and follow-up (24 months).

**Results:**

- Qualitative:
  - In participating practices (*n* = 4), dispensing with protocol encouraged reflection on tailored care and selection of various self-management interventions
  - A focus on patient preferences, team collaboration and intervention feasibility was associated with high implementation fidelity
- Quantitative:
  - In patients (*n* = 126), likelihood of complete monitoring decreased significantly after two years (OR 0.2 (95% CI 0.1–0.5), *p* < 0.001)
  - Satisfaction decreased slightly (− 1.6 (95% CI -2.6;-0.6), *p* = 0.001)
  - Non-significant declines were found in wellbeing (− 1.3 (95% CI -5.4; 2.9), *p* = 0.55) and health status (− 3.0 (95% CI -7.1; 1.2), *p* = 0.16).

**Conclusions:**

To tailor diabetes care to individual patients within well-organised practices, we recommend dispensing with protocol while maintaining one structural annual monitoring consultation, combined with the well-supported implementation of feasible self-management interventions. Interventions should be selected and delivered with the involvement of patients and should involve population preferences and solid team collaborations.

**Supplementary Information:**

The online version contains supplementary material available at 10.1186/s12913-021-07198-2.

## Introduction

**Fig. 1:**
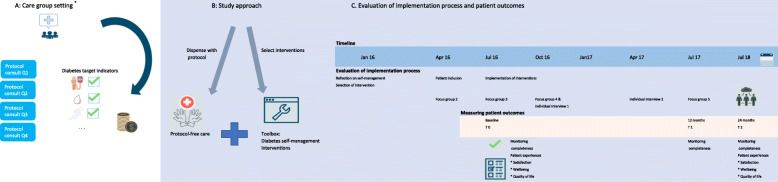
Overview of care group setting, study approach and study outcomes

Diabetes primary care is increasingly delivered based on structured care protocols [1–4]. In the Netherlands, where 6.0% of all inhabitants had a diagnosis of type 2 diabetes in 2015 [5], more than 80% of them were treated in primary care [6]. Professional guidelines for standard diabetes primary care - developed by a national scientific council for general practitioners (GPs) - include monitoring of HbA1c levels, systolic blood pressure and LDL together with lifestyle-related indicators, at least once a year [7]. To improve adherence to these guidelines, most GPs have now unified into ‘care groups’, which facilitate delivery of structured diabetes care protocols and provide logistic and quality support to individual practices [8]. For a description of the protocol and care group approach, see Textbox 1 and Fig. 1.

**Textbox 1:** Care group approach and diabetes protocol

The care group approach supports stakeholders at several levels. People with type 2 diabetes are offered a protocol comprising 3-monthly consultations at the practice location by the GP or nurse practitioner. During these consultations, the GP or nurse practitioner monitors diabetes-related health indicators and provides lifestyle coaching [9]. Generally, one annual consultation, specifically focused on monitoring of biomedical health indicators, is delivered by the GP. The additional three consultations, which are typically delivered by nurse practitioners, are primarily dedicated to lifestyle counselling and self-management support. Participation is free of charge for individuals and all consultations are reimbursed by health insurance companies. For practices, care group support includes i) the availability of a team of specialised nurses who provide coaching with regard to the implementation of protocols, ii) task delegation from GPs to nurse practitioners, iii) an electronic system providing up-to-date monitoring information on the diabetes population; and iv) professional education. In addition, care groups negotiate with health insurance companies on behalf of participating practices regarding the content of the structured care protocols, annual quality targets and reimbursements. Although quality targets and reimbursements vary depending on local agreements between care groups and insurance companies, annual quality registrations of all care groups are monitored on a national level. More specifically, all care groups are asked to provide data on the number of people with at least one registration of a predefined set of diabetes health indicators including HbA1c, systolic blood pressure, LDL and lifestyle-related variables. More details on care group support, roles and responsibilities in the practice team are presented in Additional file 1, Table 1.

Structured type 2 diabetes primary care is associated with improved monitoring of key biomedical and lifestyle-related health indicators [10, 11] and better monitoring of these indicators is associated with lower HbA1c levels [12], particularly in poorly-controlled people [13]. However, given that guideline compliance is known to be affected by physician attitudes [14], protocol-based delivery of diabetes primary care is the subject of growing discussion. For example, many GPs find protocols too restrictive [15], or insufficiently flexible and thus of limited value for individual patients [16]. In addition, a systematic metareview revealed that GPs not only experience clinical professional guidelines as undermining their professional autonomy and limiting treatment options but also doubt the credibility of underlying scientific evidence [17]. Furthermore, GPs who use care protocols report barriers such as additional registration duties and perceived bureaucracy [18], while at the same time, gaps have been reported concerning the adjustment of diabetes care to individual needs [19].

In line with the perspective of the so-called ‘patient-centered medical homes’ in the United States [20], GPs would reportedly prefer to adjust diabetes care to individual patient preferences [21], which might improve patient ‘self-management’, defined here as ‘the ability to navigate optimally through a multitude of daily disease-related decisions and care activities’ [22]. Empowerment of patient self-management is considered a cornerstone of appropriate diabetes care [3, 22–24] - particularly considering recent developments around COVID-19 [25] that hinder delivery of in-person diabetes care. Many self-management interventions are available and a national Dutch toolkit of self-management interventions [26] includes, amongst others, group-based training to improve people’s coping skills with regard to diabetes self-management, including goal-setting and problem-solving skills [27], an SMS service that healthcare professionals can use to periodically send patients messages encouraging lifestyle adjustment; and an online application in which healthcare providers can present 5-minutes blocks of information on various disease-related topics. Unfortunately, evidence for the effectiveness of self-management interventions in primary care is fairly mixed [28–31], which might be partly related to the fidelity of the implementation process, since outcomes are strongly affected by process elements such as implementation strategies, quality of delivery and participant responsiveness [32]. A refined model covering generic aspects of implementation [33] provides insight into implementation. These include A) Implementation strategies: specification of strategies used to support optimal and standardised implementation; B) Coverage: Proportion of intervention participants who received the implementation strategy; C) Participant responsiveness: The extent to which participants are engaged by and involved in the activities and content of the program; and D) Quality of delivery regarding intervention components: The extent to which the intervention is delivered in correspondence with its design. In this study, an implementation combined with sufficient attention for these process elements is classified as successful.

To our knowledge, however, little is currently known regarding the experiences of GP practices that dispense with care protocols or regarding facilitators of successful implementation of self-management interventions in primary diabetes care. Within a study setting, practices may feel that interventions are ‘time-consuming’ and ‘too disruptive’, which may hinder implementation or delivery of interventions as originally intended [34, 35]. In other words, successful implementation requires that factors related to providers and to the organisational context both receive sufficient attention [36]. Furthermore, insight into effective strategies to select interventions [37] is needed in order to overcome practice-related barriers.

While more effort is needed regarding uptake of the implementation process, it is nevertheless important to respect professional autonomy and personalised care [38]. Therefore, in the context of this study, we regard practices as experts in terms of possibilities to tailor care and in the selection of appropriate interventions in their specific population and organisational context. In our view, dispensing with protocol is relatively safe in well-organised practices that see the majority of their patients at least once a year. In view of the goal of tailored care, the primary aims of this study were explored with qualitative methods, in order to gain insight into experiences of well-organised practices regarding a) dispensing with diabetes protocol including development of a vision concerning the tailoring of care for individual patients; and b) to determine the key conditions for successful implementation of self-management interventions as a ‘proof of concept’ within well-organised practices. Furthermore, to facilitate a better understanding of patient outcomes, we investigated - on an exploratory basis - the impact of tailored care on people with diabetes concerning monitoring, satisfaction, wellbeing and health status.

## Methods

### Setting

This study was conducted among practices participating in Hadoks, formerly known as care group ELZHA, which included 157 practices in January 2016. At that time, Hadoks offered structured primary care protocols for type 2 diabetes, chronic obstructive pulmonary disease and cardiovascular disease management to socioeconomically and culturally diverse populations. On behalf of practices, annual targets for the registration of patient monitoring were negotiated with insurance companies. Socioeconomic characteristics, categorised as deprived, intermediate or advantageous, were based on standardised calculations by the municipality of The Hague [39].

### Study design

In this mixed-methods prospective study, practices were allowed to dispense with diabetes protocol and to implement self-management intervention(s) as an alternative. A qualitative case study approach [40] was used to study experiences of practices regarding dispensing with protocol and the process of implementation of self-management interventions. Furthermore, to determine experiences of people with diabetes, quantitative methods were used to measure completeness of diabetes monitoring, satisfaction, wellbeing and health status.

### Intervention

From January 2016 through July 2017, study practices were permitted to dispense with the diabetes protocol including registration duties, while maintaining reimbursements. Practices had the opportunity to choose and implement self-management interventions inspired by a nationally approved set of self-management tools [26], based on their view of the practice population and their preferences as a practice. Study participation included implementation support by KB, coordinator for the Hadoks staff nurse team, who was available for questions and general assistance. In addition, collective study meetings were organised, including development and presentation of an action plan for implementation, and the identification of barriers and facilitators affecting the implementation process etcetera, which enabled practice teams to reflect on their progress and to exchange tips and tricks. Moreover, these topics, including support needs, were discussed in more detail during the individual practice visits (see Additional file 2, Table 1). An overview of the study structure is presented in Fig. 1. From January to March 2016, practices were challenged to think about the tailoring of care to individual patients in their own practice and to subsequently choose at least one self-management intervention. From April to July 2016, practices invited patients to participate in the study. From August 2016 through July 2017, practices had the opportunity to implement the self-management interventions of their choice. From the perspective of the patients, the intervention included exposure to the self-management interventions as implemented by their practices.

### Sampling of practices and patients

According to Hadoks quality standards, practices were classified as well-organised if 1) they offered the diabetes protocol and at least one other care protocol, and 2) monitoring targets were met in calendar year 2014. Details are provided in Additional file 1, Table 2. Between October and December 2015, all well-organised practices were invited to participate – both personally by Hadoks’ staff nurses and in written form. Study practices selected adult individuals who at that point had received the diabetes protocol for at least 1 yr, had a HbA1c ≤ 64 mmol/mol and had no insulin treatment. All patients meeting these eligibility criteria were invited by their practice, in writing, to participate in the study. If necessary, a written reminder was sent after a period of 2 weeks. Patients were only enrolled when written informed consent was received.

### Data collection

#### Qualitative study

Five semi-structured focus group sessions, led by KB (health scientist and Hadoks’ staff nurse team coordinator) and SvB (psychologist skilled in qualitative research methods) were held with GPs and nurse practitioners from all included practices. Furthermore, two semi-structured individual interviews, conducted by SvB and KB, were held at each practice location. All focus groups and individual interviews were attended by each practice team, and at least one GP and one nurse practitionerwas present from each practice. A topic guide (see Additional file 2, Table 1) was used for all focus groups and interviews, which also provided room for participants to raise their own issues. Focus groups and interviews were audiotaped with the consent of the participants and were transcribed verbatim.

#### Quantitative study

To determine monitoring completeness at baseline (T0), after 12 months (T1) and after 24 months (T2), we used pseudonymised data on patient monitoring that was obtained from the primary care data registry. To gain insight into various aspects of patient experiences, several questionnaires were used which participating patients received at home immediately after study registration (T0). They were asked to complete and return the questionnaires to the university’s general support desk. If necessary, patients received a reminder after 2 weeks. Patients received follow-up questionnaires 24 months later (T2), which were also followed by a reminder after 2 weeks where necessary.

### Outcomes

#### Qualitative study

Practice level: 1) GPs’ and nurse practitioners’ experiences regarding dispensing with diabetes protocol, which were measured during focus group 1, 2 and 5; 2) vision development concerning tailored care (focus group 1 and 2) and construction of action plan for the implementation of the selected intervention (focus group 2); 3) the implementation process regarding self-management interventions, operationalised by the assessment of implementation fidelity and identification of elements essential to successful implementation, which was investigated during focus groups 2, 3, 4 and 5 and the individual practice interviews.

#### Quantitative study

Patient level: 1) the odds of patients being monitored as recommended by professional GP guidelines [7]. Accordingly, patients were defined as being ‘monitored as recommended’ if at least one measure had been registered in the previous 12 months for each of the biomedical (HbA1c, systolic blood pressure, LDL) and lifestyle-related (body mass index, smoking behaviour, physical exercise) target indicators [10, 12]; 2) Patient experiences at baseline (T0) and after 24 months (T2) as determined by the following questionnaires: A) Treatment satisfaction: Diabetes Treatment Satisfaction Questionnaire [41] (DTSQ, 1,4,5,6,7,8, total score 0 = very negative to 36 = very positive); B) Wellbeing: World Health Organization Wellbeing Index-5 [42] (WHO-5, 5-item total score 0 = very low, 100 = very high); C) Health status: EuroQol Visual Analogue Scale [43] (EQ-VAS, 1 item), score 0 = worst imaginable, 100 = best imaginable).

### Data analysis

#### Qualitative analysis

Pseudonymised transcripts of all group and individual sessions were studied independently by two researchers (SvB and JSM, master in clinical psychology). First, all transcripts were read and analysed separately based on content analysis [44]. This included, after initial exploration of the transcriptions, deductive coding based on categories that were derived from our conceptual model. In each category, emerging themes were identified. Then, in an ongoing analysis, discrepancies and disagreements that emerged were discussed with co-authors until consensus was reached. Using the final coding, a codebook for dispensing with diabetes protocol and the implementation process was constructed.

A checklist [33] which was originally developed for the assessment of implementation fidelity within studies, was subsequently applied to the codebook to assess intervention implementation as reported by practices. Each intervention was assessed from zero to maximally two points on a) fidelity of implementation strategies, b) coverage and c) participant responsiveness (for the checklist including rating details, see Additional file 2, Table 2). In addition, the quality of delivery was rated as ‘good’ or ‘limited’. The sum of all points resulting in a final rating of implementation fidelity. Components essential for successful implementation were derived from the facilitators within interventions with a high rating of implementation fidelity and from barriers within low-rated interventions.

#### Quantitative analysis

As regards patient baseline characteristics, categorical variables were reported as numbers and percentages. Continuous variables were reported as means with standard deviations (SD) or, in case of non-normal distribution, as medians with interquartile ranges (IQR). To compare odds of patients being monitored as recommended at T0, T1, and T2, logistic multilevel analysis was carried out. To compare patient satisfaction, wellbeing and health status at T0 and T2 (not available at T1), linear multi-level analyses were performed. Multilevel analysis allowed us to adjust individual observations (level 1) for GP practice (level 2). In addition, analyses were adjusted for age and diabetes duration (in quartiles), and for gender. Descriptive statistics were analysed using SPSS version 24.0. Multilevel analyses were performed using ML WiN (Version 2.28).

## Results

### Qualitative study

Of the 49 practices approached, four practices varying in size, organisation and social-economic characteristics of practice location (Table 2) agreed to participate in the study. No specific characteristics differentiated participating and non-participating practices. Participating GPs and nurse practitioners differed in age and years of experience and eExcept for one GP, all participants were female. Illustrative quotes of participants are presented in Table 3.

**Table 2:** Baseline data of participating practices

	A	B	C	D	Total
Volume of registered patients	2 * norm^1^	1.5* norm	2* norm	> 2 * norm	
Socioeconomic status	Deprived	Mixed (deprived/advantageous)	Advantageous	Deprived	
Primary intervention	SMS service	Exploration of patient needs	Patient ePortal	Consultation reduction	
Participants (n)	49	31	11	35	126
Age (years): median [IQR]	68 [61–72]	68 [64–76]	70 [59–80]	64 [62–70]	68 [62–72]
Diabetes duration (years): median [IQR]	7 [3–9]	6 [2–8]	3 [2–8]	7 [3–10]	6 [3–9]
Gender: female n (%)	21 (43%)	17 (55%)	3 (27%)	14 (40%)	55 (44%)
Monitoring as recommended, n (%)	48 (98%)	25 (81%)	11 (100%)	31 (89%)	115 (91%)
DTSQ Status^2^: mean (SD)	30.8 (6.5)	32.3 (3.9)	31.3 (6.0)	29.6 (5.4)	30.9 (5.6)
WHO-5: mean (SD)	54.7 (25.0)	68.2 (15.5)	66.5 (26.4)	53.9 (22.9)	58.4 (23.3)
EQ-VAS: mean (SD)	65.3 (22.2)	77.8 (16.6)	82.8 (11.1)	65.8 (16.5)	69.5 (17.7)

Abbreviations

DTSQ: Diabetes Treatment Satisfaction Scale

WHO-5 = World Health Organization Wellbeing Index-5

EQ-VAS: EuroQol Visual Analogue Scale

SES: socioeconomic status

^1^National norm for average practice volume: 2095 patients.

^2^DTSQ: Status = all items except no 2 and 3

**Table 3:** Interpretation and scoring of implementation fidelity elements for interventions in each practice (see separate file)

	Practice A		Practice B		Practice C		Practice D		Emerging themes
Experiences of dispensing protocol	**A1.1 FG 5, NP:** The liberating part of this project is that you can think: “this year I don’t get judged.” So that lowers the bar. Yes, I am in favour of dispensing with protocol, but not when I will be judged on it eventually.		**B1.1, FG 5, NP:** Well it provided the impetus to start conversations with people in a different way. (…) Yes, [we have] developed some more contact with other disciplines in the neighbourhood. And yes, indeed [when you] get started,. you get thrown in at the deep end.		**C1.1, FG 5, NP**: But because we could be independent of numbers (…) you get a different perspective, and a different focus. Now we can focus on self-management.		**D1.1, FG 5, NP:** I have often asked you what we would do with it. So we were not sure what it would entail and how it would continue. It was a bit of a wait.		-Liberty facilitating room for an approach more tailored to individual patients -Confusion concerning expected delivery of care
Vision on tailored care	**A2.1, PI 2, GP**: It might sound trivial, (…) but if they previously never showed up and now they do, then that is already a win.		**B2.1, FG 2, GP:** If the goal is to stimulate self-management and control in the patient, then the starting point is totally wrong if we decide what the patient has to work with. (…) Patients need to be able to make this choice themselves.		**C2.1, PI 1, GP:** Just that [personal aims related to diabetes] already, that people start to think about it at home, fill it in and write it down, then we have gained a lot already. **GP B:** Then you can provide much more targeted information.		**D2.1, FG 2, NP:** Actually, dispensing with protocol [is good] for people who have to come twice a year at most, who are doing fine and are taking responsibility (…). I am very happy with this project. [Besides that] I will not be pushing the unwilling anymore. If they don’t want to, then don’t. There’s plenty of people who do want to and who are worth the energy investment.		- Improvement of protocol compliance - Shifting care to patient preferences -Encouraging patient involvement
Interven-tion	**SMS reminder service**		**Layered exploration of patients’ needs**		**Patient e-portal**		**Consultation reduction**		
	***Implementation fidelity element including rating (0 = low, 2 = high)***								***Emerging themes***
Strategies^1^	**A.3.1 PI 1, NP:** The system is very easy. (…) We encountered some problems (…). Often, mobile phone numbers were not saved in the right place in the electronic patient record, and then the SMS service would not get linked to it. (…) [we worked on this with] the whole team: if someone shows up at the front desk, ask them whether they have a cellphone number and then check whether it is saved in the right place. (…). So it does have a sort of start-up phase (…). You really have to be dedicated (…) So we are already paying attention to it as much as possible. **A.3.2 PI 1, NP:** And I have to check: How much time does this cost? And thenI possibly [have to] cancel a consultation so that I have more time for that.	2	**B3.1, FG 2, GP**: We started thinking: how can we do this? (…) To approach a few project participants to attend an externally organised sort of meeting at the practice (…), that was our first step (…). The second step was that we wanted to invite the entire group of participants (…) to provide information about which self-management tools wewould offer as a practice (…) to these patients, and then see if people were keen (…). So we are still in the phase where we don’t know what we will do at all. We will see. I’m curious. **B3.2, PI 1, GP:** Regarding our choice in favour of a patient portal, I think that we should give ourselves enough time (…) I think that it will be “yes”, but I think that this needs to be a practice-wide decision.	2	**C3.1, PI 1, GP:** The primary aim is about putting the patient in control, with eVita as a means to make patients do their homework (…) That is the essence of eVita. So we expect a lot from this. **C3.2, FG 2, GP:** The user’s manual for eVita has to be so simple that (…) you can explain everything on single sheet of paper. (…) There will be patients who do not know how to use a computer. They might get a notification: “Write it down [on paper]” and then you have already achieved something. That has to be possible too.	2	**D3.1 PI 2, NP:** We told a lot of people that they were doing fine and that visiting four times a year was unnecessary;. that once a year was also fine.	0	-Involvement of practice team -Consideration of patient preferences -Communication with patients
Coverage^1^	**A4.1, FG 4, GP**: We can now invite people by SMS. And [having started with the study participants], we now want to extend this to all nurse practitioners and all of our diabetes patients.	2	**B4.1, FG 2, GP:** One is more articulate than the other in the practice (…) **FG 5, NP:** We invited four patients to join the patient panel. **B4.2, PI 1, GP:** A kind of patient meeting where we send a message to all diabetics. Kind of an open invitation (…). Maybe the physical therapist can give some more information. Maybe the dietician can join in. Just to give it some features, raise its profile a bit. **B4.3, FG 5, NP:** We sent by post.invitation letters fconcerning the health market to 230 patients	2	**C4.1, FG 5, NP:** Based on your inclusion criteria, 90 patients were eligible [in our entire T2DM-population] and 33 signed up. 15 people actually used it. **GP**: And 10 actually logged in.	1	**D4.1, FG 3, NP 2:** I feel like I should only let the motivated people take part, otherwise it is just a constant up hill struggle (…) Some say: “Maybe.” Then I think: Well, this one is not motivated.	1	Not applicable
Participant responsiveness^1^	**A5.1**, **FG 5, NP:** Patients always ask “Will I get a text message again next time? Because I really appreciate it.” (…) Other people are like “well if you hadn’t sent that text, I wouldn’t have come.” (…) You can see that patients do really appreciate it.	2	**Patient panel:** **B5.1, PI 1, NP:** Look, obviously it was a very small group, but I am very happy with what has come out of it. **FG 5, NP:** People have often told me: “We thought it was a really nice evening, because you could share experiences with each other.” **Health market:** **B5.2, FG 5, NP:** It was in the late afternoon. But a Thursday or a Friday? (…) Also neighbourhood-wide (…). I think about seventy came. There were fifty who filled in the evaluation forms. Five or six patients signed up for eVita at the time, but now, I have got three additional registrations. (…) Nine people also registered for a course about ‘Living with diabetes’ (…) Three nights of two and a half hours, for a maximum of 12 people. **B5.3, FG 5, NP:** Yes, but afterwards we did hear from people “it was great fun, you should do this more often!” There were also people wo said: “Well... that wasn’t really necessary.” It gave a boost to do something like this again.	2	**C5.1, FG 5, NP:** Even if you say “This is eVita, you can enter your improvement goals here,” people still need guidance. (…) That it is of no use to them if you say “Okay, we figured it out: you actually have four goals of improvement, now get to work to see which ones you want to work on and then figure out how you want to do that (…). It is really letting the patients decide for themselves: “Well we have four things that stand out, what would you like to work on? And shall we write that down as a goal for improvement? Then we get back to that the next time.” That is really what works (…) People really have to be motivated and you have to lead them by the hand to maintain self-management. **C5.2, FG 5, NP:** No, and not everyone was equally enthusiastic about eVita. Many people felt it was patronising.	1	**D5.1, FG 5, NP:** Well yeah, you may not want them to visit, but still they want to come. [It must give a feeling] of safety, familiarity. [They are] scared too, that if they don’t visit for a year, it gets a lot worse all of a sudden. What then? So for some patients, it was quite difficult not to have to come anymore.	1	-Variability in response of patients
Quality of delivery^2^	**A6.1, PI 2, NP:** First, I created a text message group, which was much faster. But then if someone cancels you can’t remove that person from the group. I find that very patient unfriendly. You can’t do that. (…) Then people get confused “I thought I cancelled?”	+	**B6.1, FG 5, NP:** Last year was one of the first steps (…) [creating] a patient panel (…). We wanted to keep it neutral, [so] we were not present ourselves. (…) Different things were brought up. (…) For example, the need to look up information and blood results (…), a diabetes course, advice about food (…) and exercise (…) As a result, we organised ahealth information market (…). A range of disciplines of the local area participated (…) Although everyone focused on diabetes care, some also covered care for the elderly.	+	**C6.1, PI 2, NP:** In my opinion, eVita is not yet where it has to be. (…) I don’t think it is very clear, it is a bit abracadabra. That is also the feedback I get from people. (…) Well some [already encounter problems] upon signing up, but then you have problems really early on. I had a man in here twice saying: (…) “I really want it, but I just can’t do it”. (…) [In contrast to the desktop version], the [mobile] app only allows the input and display of certain predetermined values. And there you can’t see the videos. That’s a pity. **C6.2, PI 2, GP:** And those videos were pretty stupid.	–	**D6.1, PI 3, NP:** I feel like (…)we didn’t keep going. (…) A person with diabetes attends your consultation hour and our system then states says “Participating in the project.” But the program is not any different. At least, with the people I see, I do the same things I always do. **D6.2**, **PI 3, NP:** No, nothing has changed. **NP:** I think that some people may have visited less often, but I don’t have an overview of that.	–	-Sensitivity to patients’ needs - Involvement of practice team -Negative experiences concerning user-friendliness of the ePortal
Implemen-tation fidelity: sum score	6		6		4		2		
General fidelity	High				Low				

#### Experiences concerning dispensing with diabetes protocol

Three practices had positive experiences concerning dispensing with diabetes protocol. In practice A, a sense of freedom was reported. “The liberating part. .. is that you think: ‘This year, I don’t get judged’. So that lowers the bar,” (Table 3, #A1.1). According to practice B, ‘it provided the impetus to start conversations with people in a different way,’ (Table 3, #B1.1). Both experiences came together in practice C, “Because we could be independent of numbers. .. you get a different perspective. . ., can focus on self-management,” (Table 3, #C1.1). Practice D primarily experienced a lack of clarity about what to do: “We were not sure what it would entail and how it would continue, it was a bit of a wait,” (Table 3, #D1.1). Key themes can be characterised as *liberty facilitating a more person-centred approach* versus *confusion*.

#### Vision development on tailored care and selection of self-management interventions

The process of reflection on the tailoring care to individual patients resulted in a disparity of views across the participating practices. Practice A, where the level of non-attendance was high, aimed at supporting patients in order to improve consultation attendance: “It might sound trivial. .. but if they [previously] never showed up and now they do, then that is already a win,” (#A2.1). This resulted in the selection of an SMS reminder service to help patients remember their diabetes consultation.

Practice B stated that patients should have an important voice in the development of care tailoring. “… The starting point is totally wrong if we decide what the patient has to work with. .. Patients need to be able to make this choice themselves,” (#B2.1). Subsequently, they developed a layered approach to exploring patients’ preferences.

In the view of practice C, tailoring of care meant adapting the consultation to a patient’s information needs, “… That people start to think about it at home. .. then you can provide much more targeted information,” (#C2.1) Therefore, a patient ePortal was selected for implementation.

Practice D perceived tailoring of care as investing in the people willing to receive diabetes care with a frequency adjusted to the patient’s wishes, in preference to investing in people with little motivation. “Actually, dispensing with protocol [is good] for people. .. who are doing fine and taking responsibility. [Besides that] I will not be pushing the unwilling anymore. .. There‘s plenty of people. .. who are worth the energy investment (#D2.1).

Amongst the multiplicity of views on tailored care, several themes were observed that could be refined to ‘*improvement of protocol compliance’, ‘shifting care to patient preferences’* and *‘encouraging patient involvement’*. These different themes were mirrored in the varied choices of self-management interventions, which were primarily patient-focused, such as the SMS reminder service, explicit exploration of patient needs with subsequent selection of instruments, and the ePortal, or, in the case of consultation reduction, practice-focused (Additional file 2, Table 3).

#### Implementation process: conceptual elements of implementation fidelity

##### Implementation strategies

The applied implementation strategies could be broadly differentiated. For example, although the implementation of the SMS service for patients in practice A appeared relatively straightforward, it still required changes regarding registration procedures and information sharing within the entire practice team, including medical assistants. “We encountered some problems. .. [We worked on this] with the whole team. .. So it does have a sort of start-up phase.. .. You really have to be dedicated,” (#A3.1). Practice B decided to consult a representative patient panel concerning their preferences regarding self-management interventions. Subsequently, this practice presented the panel’s recommendations to all patients with diabetes registered at their practice during a large-scale health event known as a ‘health market’, with the aim of implementing popular interventions. “To approach a few project participants to attend an externally organised sort of meeting at the practice... , that was our first step. The second step was to invite the entire group of participants to provide information about which self-management tools we would offer as a practice. .. and then see if people were keen,” (#B3.1). Furthermore, concerning the selection of concrete interventions, the commitment of the full practice team was important. “Regarding our choice. .. I think it will be a yes but I think that this needs to be a practice-wide decision,” (#B3.2).

Practice C decided to implement the ePortal for patients while providing support with an easily-accessible instruction guide. “The user’s manual has to be so simple that you can explain everything on a single sheet of paper,” (#C3.2). Practice D did not report actually considering of patients’ preferences, but simply offered a reduction of consultation frequency within a framework of standard diabetes consultations. “We told a lot of people that they were doing fine and that visiting four times a year was unnecessary; that once a year was also fine,” (#D3.1). Key themes that emerged concerning implementation strategies included *involvement of the practice team*, *consideration of patients’ preferences* and *communication with patients*.

#### Coverage

Practice A, B and C targeted their interventions to all the diabetes patients in the practice. Practice A: “We can now invite people by SMS. And [having started with the study participants] we now want to extend this to all nurse practitioners and all of our diabetes patients,” (#A4.1). Practice B: “We invited four patients to join the patient panel,”(#B4.1). “We sent by post information letters concerning the health market to 230 patients (#B4.3). Practice C:” Based on your inclusion criteria, 90 patients were eligible and 33 signed up,” (#C4.1). Practice D focused exclusively on motivated patients amongst the study participants. “I feel like: I should only let the motivated people take part, otherwise it is just a constant up hill struggle,” (#D4.1).

#### Participant responsiveness

Participant responsiveness was high in practice A, where patients actively requested continuation of the SMS service. “Patients always ask, ‘Will I get a text message again next time?. .. Other people are like ‘Well if you hadn’t sent that text, I wouldn’t have come’,” (#A5.1). The layered approach chosen by practice B was also very positively received, by patients as well as by the practice team itself. “Look, obviously it was a very small group, but I am very pleased with what has come out of it. People have often told me: ‘We thought it was a really nice evening, because you could share experiences with each other,” (#B5.1). Furthermore, the health market was well-attended. “It was in the late afternoon. I think about seventy came.. .. Five or six patients signed up for eVita at the time, but now I have three additional registrations. Nine people also registered for a course about ‘Living with diabetes’,” (#B5.2). There was an overall good response from patients– which in turn resulted in enthusiasm among the practice team. “It gave a boost to do something like this again,” (#B5.3).

In practice C, patients apparently needed more than a user manual to be able to use the ePortal. “Even if you say: ‘This is eVita, you can enter your improvement goals here’, people still need guidance.. .. People really have to be motivated and you have to lead them by the hand to maintain self-management,” (#C5.1). In addition, the enthusiasm of patients was limited. “Many people felt it was patronising,” and participant responsiveness was consequently limited (#C5.2). In practice D, patients’ willingness to reduce consultation frequency was low for reasons of safety and fear of worsening diabetes health, “Well yeah, you may not want them to visit, but they still want to come. [It must give a feeling] of safety, familiarity; [they are] scared too, that if they don’t visit for a year, it gets a lot worse all of a sudden,” (#D5.1). Thus, across the participating practices, the responsiveness of patients to the selected interventions varied considerably.

#### Quality of delivery

The SMS service in practice A was delivered with high sensitivity from the perspective of patients. “First, I created a text message group, which was much faster. But then if someone cancels you can’t remove that person from the group. I find that very patient-unfriendly. You can’t do that. ... Then people get confused; “I thought I cancelled?’” (#A6.1). The layered exploration of patient needs by practice B was also characterised by thorough delivery in agreement with its initial goal, “Last year was one of the first steps... [creating] a patient panel. .. Different things were brought up... For example, the need to look up information and blood results ( …), a diabetes course, advice about food.. and exercise. .. As a result, we organised a health information market. .. A range of disciplines from the local area participated. .. Although everyone focused on diabetes care, some also covered care for the elderly,” (#B6.1).

In the other practices the quality of intervention delivery was limited. Implementation of the ePortal by practice C was not yet feasible since patients reported that the ePortal was complicated to use. “In my opinion, eVita is not yet where it has to be.. .. That is also the feedback I get from people.. .. Well some [already encounter problems] upon signing up, but then you have problems really early on. I had a man in here twice saying. .. “I really want it, but I just can’t do it”.. .. [In contrast to the desktop version], the [mobile] app only allows the input and display of certain predetermined values. And there you can’t see the videos. That’s a pity,” (#C6.1). Furthermore, the tutorial clips were perceived as low-quality, “And those videos were pretty stupid,” (#C6.2). In practice D, the plan to reduce consultations had simply not been implemented and no differences in daily care delivery were reported. “I feel like. .. we didn’t keep going.. .. A person with diabetes attends your consultation hour and our system then states: “Participating in the project.” But the program is not any different. At least, with the people I see, I do the same things I always do. .. I think that some people may have visited less often, but I don’t have an overview of that,” (#D6.1). In other words, there was no perceived delivery of consultation reduction. The themes that emerged regarding quality of delivery included *differing sensitivity to patients’ needs and preferences*, *involvement of the practice team* and *negative experiences regarding user-friendliness of the ePortal.*

#### Rating of implementation fidelity and identification of essential components

Implementation fidelity in practice A and B (overall score: 6) was rated as high, but was limited in practice C (score: 4) and D (score: 2) (Table 3). As three practices reported that dispensing with protocol encouraged new ideas regarding changes to care and stimulated out-of-the-box reflection on appropriate interventions, this was identified as the first essential component for successful implementation of self-management interventions.

Practices A and B, both of which had with high implementation fidelity, were characterised by high sensitivity to patient needs and preferences (see #A6.1 and #B2.1) and a strongly collaborative team (see #A3.1 and #B3.2). As the implementation of the patient ePortal by practice C demonstrated, interventions should first be adjusted to users’ needs before implementation. In practice D, a lack of focus on people’s needs coincided with limited development of a vision on patient-centred care. To summarise, development of a consistent view on the tailoring of care that is rooted in awareness of people’s needs and preferences, together with suitable implementation strategies, was of crucial importance for successful implementation.

Developing this view and successful care tailoring can be supported by the conceptual framework. With regard to this framework, an association between intervention and outcomes is depicted. This association is affected by the previously described implementation elements and the implementation level of fidelity. The key conditions for successful care tailoring that arose from our results, can be positioned before the intervention itself. Firstly, a flexible protocol that enables reflection on suitable instruments to tailor care, is necessary prior to the actual choice of interventions. Subsequently, when interventions are selected with sensitivity for patient needs, solid team collaboration and feasibility in practice, the varying elements that constitute the implementation fidelity will result in optimal tailoring of care.

### Quantitative study

**Fig. 2:**
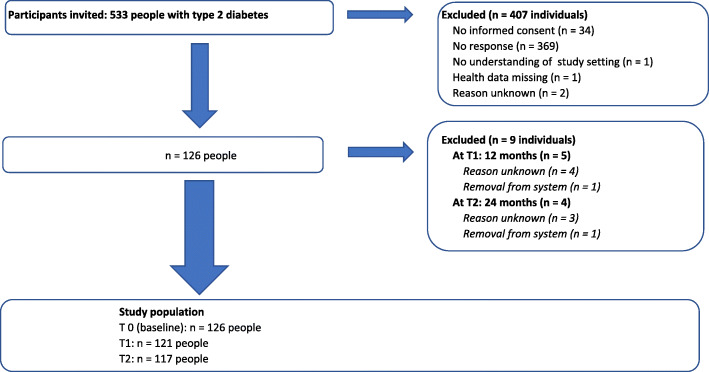
Flowchart of inclusion

Of the 533 eligible patients within the four participating practices, 24% (*n* = 126 patients) provided informed consent (Fig. 2). Loss to follow-up was 4% at T1 (*n* = 5 patients), and an additional 3% at T2 (*n* = 4 patients). Patient outcomes (diabetes monitoring, satisfaction, wellbeing and health status) at T0, T1 and T2 are presented in Table 4. With regard to monitoring, adjusted analyses showed that patients were less likely to remain monitored as recommended, with a non-significant difference at T1 (OR 0.7 (95% CI 0.3–1.5), *p* = 0.34, see Table 5) and a significant difference at T2 (OR 0.2 (95% CI 0.1–0.5), *p* < 0.001), compared to T0. Patient satisfaction with diabetes treatment at T2 was slightly lower compared to T0 (− 1.6 (95% CI -2.6;-0.6), *p* = 0.001). For wellbeing (− 1.3 (95% CI − 5.4;2.9), *p* = 0.55) and health status (− 3.0 (95% CI − 7.1;1.2), *p* = 0.16), no significant differences were observed between T0 and T2.

**Table 4:** Patient outcomes at baseline, 12 and 24 months

Measure	T0 (baseline) (***n*** = 126)	T1 (***n*** = 121)	T2 (***n*** = 117)
Monitoring as recommended, n (%)	115 (91%)	106 (88%)	84 (72%)
DTSQ Status: mean (SD)	30.9 (5.6)	N/a^1^	29.2 (5.1)
WHO-5: mean (SD)	58.4 (23.3)	N/a^1^	56.2 (23.5)
EQ-VAS: mean (SD)	69.5 (19.7)	N/a^1^	66.6 (19.2)

Abbreviations

DTSQ: Diabetes Treatment Satisfaction Scale

WHO-5: World Health Organization Wellbeing Index-5

EQ-VAS: EuroQol Visual Analogue Scale

^1^ N/a: not available.

**Table 5:** Multi-level analysis evaluating the difference at T1 and T2 compared to T0 (baseline)

	T1				T2			
	Crude		Adjusted^**1**^		Crude		Adjusted^**1**^	
	OR (95% CI)	p	OR (95% CI)	p	OR / B (95% CI)	p	OR / B (95% CI)	p
Monitoring as recommended (OR)	0.7 (0.3–1.5)	0.35	0.7 (0.3–1.5)	0.34	0.2 (0.1–0.5)	< 0.001	0.2 (0.1–0.5)	< 0.001
DTSQ-Status^2^ (B)	N/A^2^		N/A		−1.8 (−2.8;-0.8)	< 0.001	− 1.6 (− 2.6;-0.6)	0.001
WHO-5^4^ (B)	N/A		N/A		−1.3 (−5.5;2.8)	0.53	−1.3 (− 5.4; 2.9)	0.55
EQ-VAS^5^ (B)	N/A		N/A		−3.0 (− 7.1;1.2)	0.16	− 3.0 (− 7.1; 1.2)	0.16

Abbreviations

DTSQ Status: Diabetes Treatment Satisfaction Scale (all items except no. 2 and 3) WHO-5: World Health Organization Wellbeing Index-5

EQ-VAS: EuroQol Visual Analogue Scale

^**1**^ Analysis adjusted for age, duration of diabetes, and gender.

^2^ N/A: not available.

## Discussion

This study had a number of goals, including the use of qualitative methods to explore the experiences of well-organised GP practices when dispensing with diabetes protocol, vision development concerning the tailoring of care to individual patients, identifying key conditions for the successful implementation of self-management interventions in primary diabetes care, and exploratory measurement of patient outcomes.

In terms of the conceptual framework, the freedom to dispense with the care protocol enabled practices to develop their own vision on self-management, subsequently affecting the choice of interventions and the fidelity of the implementation process. As illustrated by our findings, the interventions chosen by practices to help patients in optimally navigate life with diabetes, varied substantially and were not only targeted at the patient population, but sometimes also to the practice itself. This demonstrates that interventions targeted at self-management support can take many different forms. Generally, we observed a high level of commitment regarding the implementation process. In addition, a clear focus on the individual needs and preferences among the practice’s own patient population, solid team collaboration and intervention feasibility were identified as crucial factors underlying successful implementation. The importance of these factors was confirmed by their absence in one practice where a lack of focus on patients’ needs and team collaboration resulted in early abandonment of attempts to tailor care. In other words, careful consideration of these conditions enabled selection of appropriate interventions and a thorough process of implementation with consideration of all implementation components, resulting in successful tailoring of care.

To the best of our knowledge, clinicians’ professional experiences when not limited to treatment protocols have not yet been systematically investigated. Nevertheless, considering previously reported barriers with regard to protocol compliance, a less rigid protocol can be recommended. A more flexible protocol should be tailored to specific groups, including individuals needing support in order to obtain appropriate diabetes outcomes [45]. Considering that adherence to professional treatment protocols is associated with better diabetes knowledge among care providers [46] and with improved processes of care [47], we would advocate finding a balance between the benefits of these protocols and protocol-free care. Factors facilitating the application of protocols include a short and simple presentation, recommendations that require minimal resources before implementation and the involvement of end-users in the development, implementation and testing of guidelines [17].

Adjusting care in order to better match patients’ preferences is recommended internationally [20, 48, 49] and accords with previously defined strategies to involve patients in the implementation effort [50]. Although self-management interventions primarily aim to improve self-management among patients, factors to the practice itself also emerged as relevant to successful implementation. By dispensing with protocol and allowing a free choice of interventions, recognised barriers to the delivery of self-management interventions might have been overcome [34]. Together with a firm, team-based view on self-management that is rooted in the needs and preferences of the patient population, strong team collaboration confirms previously reported strategies designed to build a coalition of partners in the implementation effort [50]. Sufficient intervention feasibility might also be obtained through co-creation with the involvement of users [51]. Our findings may also contribute to a shift, from the perspective of the care provider, towards the more active involvement of patients in their own care [52], and thus represent an important step towards patient-centred care [53, 54].

In terms of the exploratory quantitative findings, we found significantly lower odds that people maintained recommended monitoring 2 years later. A decreased monitoring completeness following departure from protocol accords with data from recent, large-scale studies which found associations between financial incentives and quality-of-care measures in primary chronic care [55, 56]. Patient satisfaction, wellbeing and health status showed little or no significant declines over a two-year period. Despite satisfaction with many of the implemented measures, the small decline in patient satisfaction is in line with previous studies which found that patients with diabetes were slightly more satisfied with a higher annual consultation frequency [57]. In addition, appropriate monitoring is associated with better HbA1c levels [12]. This suggests that when dispensing with diabetes protocol, surveillance should still include at least one annual ‘monitoring consultation’ but this should be adjusted to patients’ needs. However, it should explicitly be noted that these analyses had an exploratory character and further studies are necessary to achieve a deeper understanding of patient outcomes.

This study had several strengths and limitations. A key strength of this study was the mixed-methods observational setting, which avoided any interference with the dynamics of daily GP practice and enabled inclusion of experiences from practice professionals and patients. Secondly, triangulation of researchers’ background including social scientists, health scientists and practicing GPs, together with team validation [58], improved the understanding and interpretation of our findings. Thirdly, considering that little is known about the gains when care providers are guided by – rather than limited to – treatment protocols, within this study, we aimed to provide greater dclarity on the impact of a departure from protocol and the tailoring of care on care providers. Moreover, besides our findings concerning the tailoring of care in practices, this study also provided unique initial insights into actual patient experiences when exposed to tailored care.

Some limitations also deserve mention. With regard to our qualitative study, the actual number of participating practices was relatively low and insight in reasons for non-participation is missing, which might hinder the generalisability to other well-organised practices. Nevertheless, in the midst of competing priorities in daily GP practice, non-participation might be explained by a low sense of urgency regarding self-management [34]. Furthermore, the diversity of the participating practice contributed to the reliability of our qualitative findings. Concerning our quantitative study, firstly, the design of our quantitative arm did not allow for causal inferences. Secondly, in terms of monitoring completeness of patients, a missing registration does not by definition imply that care was not provided. Thirdly, as clinical outcomes were not included, it is unclear how participant’s diabetes-related health parameters have developed – although we know from existing work that recommended monitoring generally is associated with better HbA1c levels [12]. Moreover, the generalisability of our quantitative analyses is limited due to the small number of patient participants, an obstacle that also precluded deeper quantitative analysis comparing individual practices or interventions.

As regards future research, we recommend exploring how practices can develop a team-based view on the needs of people with diabetes, how team collaboration can be improved, and how practices can implement self-management interventions without losing sight of patients’ diabetes health indicators. Moreover, to deepen our understanding of patient experiences in the context of patient-centered medical homes, it might be interesting to further explore clinical outcomes such as HbA1c levels, treatment satisfaction and, for example, consultation frequency, preferably comparing individual practices, interventions and level of implementation fidelity.

To summarise, our study shows that well-organised GP practices experience shift away from diabetes protocol as liberating and encouraging reflection on tailored care. A focus on patient needs, solid team collaboration and intervention feasibility are all crucial for successful implementation of self-management interventions in diabetes primary care.

In the context of COVID-19, tailoring of care to individual patients is essential to reducing the negative impact of protocol departure on structural monitoring of individual patients. Therefore, when dispensing with diabetes protocol, we recommend maintaining one structural annual monitoring consultation, together with the implementation of feasible self-management interventions - selected and delivered with a focus on patients’ preferences and solid team collaboration. This approach can potentially lead to feasible tailored diabetes care, delivered by highly committed practice teams, with optimal empowerment of diabetes patients.

## Supplementary Information

**Additional file 1.**

**Additional file 2.**

## Data Availability

The data sets generated and analysed for the current study are not publicly available due to administrative reasons, but are available from the corresponding author on reasonable request.
