# Supplementary material for: Experiences with tailoring of primary diabetes care in well-organised general practices: a mixed-methods study
Source: BMC Health Serv Res. 2021 Nov 9;21:1218. doi: 10.1186/s12913-021-07198-2 (PMC8577855; doi:10.1186/s12913-021-07198-2)
Supplement: Supplementary file 1 — Additional file 1. [file 12913_2021_7198_MOESM1_ESM.docx]

**Appendix 1 – details on Dutch diabetes care and well-organised practices**

Table 1. Aims and components of the care group approach

| **Aim** | **Service** | **Details** |
| --- | --- | --- |
| Delivery of care | | |
|  | Care protocol | 3-monthly patient consultations at the practice location, with options for monitoring of biomedical and lifestyle-related diabetes parameters.  The physician bears responsibility for the quality of care and generally conducts one annual consultation personally. The other three consultations are typically performed by nurse practitioners. Participation is free of charge for individuals and all consultations are reimbursed by health insurance companies. |
|  | Computerised clinical decision-making support system (CCDSS) | A system that provides a real-time overview of monitoring information for each patient. Monitoring information includes: a) most recent diabetes measures (such as HbA1c level, systolic blood pressure and body-mass index), and b) an alert when available information is no longer up-to-date. |
|  | Quality support of patient monitoring | Based on the monitoring information registered in the CCDSS, barriers to delivery of care and other obstacles may be highlighted *(examples include internal obstacles related to the quarterly invitation of patients or a high ‘no-show’ rate due to socioeconomic vulnerability/ limited diabetes awareness).*  Tailored support is delivered or coordinated by the Hadoks staff nurse to help practices overcome these barriers. |
| Stimulating maintenance of up-to-date diabetes-related knowledge and skills | | |
|  | Program of relevant vocational courses adjusted to the needs of physicians and nurse practitioners | Each year, an expert team of general practitioners and staff nurses - both specialised in type 2 diabetes - selects vocational diabetes courses that meet the needs of practices participating in the care group – generally, practices with an active focus on structured diabetes care. Based on the expert-based selection of courses, the care group develops a vocational course program for participating practices. Vocational courses can include ‘medical’ themes (such as new HbA1c medication) or lifestyle-related themes (such as smoking cessation).  For physicians and nurse practitioners, attending part of the program is mandatory. |
| Organisation of care | |  |
|  | Coaching by staff nurse | - Delegation of care from physician to nurse practitioner  - Team collaboration between physicians, nurse practitioners and medical assistants  - On-the-job tailored teaching based on personal needs and preferences of practice team |
|  | Collaboration with other local disciplines | Organisation of educational or prevention-related events for diabetes patients, tailored to local population needs, in cooperation with other disciplines in the neighbourhood such as dieticians, lifestyle coaches and community workers. |
| Negotiations with healthcare insurance companies on behalf of participating practices | | |
|  | Quality control | - Determination of indicators that are clinically relevant and that reflect delivery of diabetes care  - Determination of targets with regard to the proportion of patients being monitored for these indicators |
|  | Reimbursement of care | - Tariffs concerning primary care services  - Reimbursement of costs related to additional care services supporting primary diabetes care, such as dietician counseling and smoking cessation coaching |

Table 2. Requirements for well-organised practices

| **Delivery of care protocol ^1)^** | | **Monitoring targets** *(at least one measure in calendar year 2014)* |
| --- | --- | --- |
|  | Type 2 diabetes | |
|  |  | MDRD: 90 % |
|  |  | Foot examination: 80 % |
|  |  | Fundus examination: 80 % |
|  |  |  |
|  | Chronic obstructive pulmonary disease | |
|  |  | Registration of smoking status: 80 % |
|  |  | Registration of functioning/health status (MRC or CCQ): 70 % |
|  |  |  |
|  | Cardiovascular risk management | |
|  |  | Systolic blood pressure: 80 % |
|  |  | LDL profile: 80 % |
|  |  | Registration of smoking status: 70 % |

Abbreviations: MDRD: Modification of diet in renal disease; LDL: Low-density lipids

**^1)^** Type 2 diabetes and at least one additional protocol
