# Supplementary material for: Experiences with tailoring of primary diabetes care in well-organised general practices: a mixed-methods study
Source: BMC Health Serv Res. 2021 Nov 9;21:1218. doi: 10.1186/s12913-021-07198-2 (PMC8577855; doi:10.1186/s12913-021-07198-2)
Supplement: Supplementary file 2 — Additional file 2. [file 12913_2021_7198_MOESM2_ESM.docx]

**Appendix 2 – Materials of the qualitative study**

*Table 1. Topic list for each focus group and each interview with participating GP practices*

| **Date** | **Theme** | **Topics** |
| --- | --- | --- |
| Jan 16 | Focus group 1:  Reflection and vision regarding development of tailored care | - Views on the opportunity to leave the structured diabetes care protocol - Ideals regarding diabetes care - The meaning of diabetes-related self-management in participating practices - Room for additional discussion points |
| Apr 16 | Focus group 2:  1) Dispensing with protocol  2) Aims regarding tailoring of care | - Experiences of dispensing with current protocol - Objective of participating practices - Selection of target population - Choice of self-management interventions for implementation - Action plan for implementation of selected interventions - Identification of potential facilitators or barriers regarding the implementation process, including incorporation of these factors into the action plan - Room for additional discussion points |
| July 16 | Focus group 3:  General monitoring of implementation process of self-management interventions | - Progress of implementation process in participating practices - Identification of intermediate facilitators or barriers - Needs for support (practical, logistic, general coaching) from the project team - Room for additional discussion points |
| Oct 16 | Focus group 4:  General monitoring of implementation process | See description focus group 3 |
| Oct 16 | Practice interviews, round 1:  Monitoring of implementation process in individual practices | - Progress of implementation process in participating practices - Identification of new intermediate facilitators or barriers - Needs for support (practical, logistic, general coaching) from the project team - Room for additional discussion points |
| April 17 | Practice interviews, round 2:  Monitoring of implementation process in individual practices | See description practice interviews round 1 |
| July 17 | Focus group 5:  Reflection on dispensing with protocol and tailoring of care: | - Experiences of dispensing with protocol in participating practices - Overview of selected interventions in each practice - Reflection on the implementation process and its outcomes - Observed barriers and facilitators of the implementation process - Evaluation of benefits resulting from practice participation in this project - Room for additional discussion points |

*Table 2. Checklist for assessment of implementation fidelity*

| **Element** | **Description** | **Conditions** | **Scoring** |
| --- | --- | --- | --- |
| Implementation strategy | |  |  |
|  | Specifying **the implementation strategy**(s) *and* evidence of the extent to which this/these implementation strategy(s) took place | 1: Does the practice describe all implementation strategies used? AND  2: Does the practice provide detail on how all implementation strategies were carried out? | 2 |
|  |  | 1: Does the practice describe some but not all implementation strategies used? AND  2: Does the practice provide detail on how some but not all implementation strategies were carried out? | 1 |
|  |  | 1: Does the practice describe all or some implementation strategies used? OR  2: Does the practice provide detail on how all or some of the implementation strategies were carried out? | 0^a^ |
| Coverage | |  |  |
|  | Proportion of intervention **participants** who received the implementation strategy(s) | 1: Does the practice provide a description of the number of people receiving all of the implementation strategies? AND  2: Does the practice provide a description of the strategy or strategies all of the groups received? | 2 |
|  |  | 1: Does the practice provide a description of the number of people receiving some but not all of the implementation strategies? AND  2: Does the practice provide a description of the strategy or strategies for some but not all of the groups? | 1 |
|  |  | 1: Does the practice provide a description of the number of people receiving some or all of the implementation strategies? OR  2: Does the practice provide a description of the strategy or strategies for some or all of the groups? | 0^a^ |
| Participant responsiveness | |  |  |
|  | The extent to which participants are  engaged by and involved in the activities and content of the program | 1: Does the practice state participants’ involvement in the development, evaluation, or receptivity to the implementation strategy? AND  2: Does the practice provide a description of the extent of participant involvement in the development, evaluation, or receptivity to the implementation strategy? | 2 |
|  |  | 1: Does the practice provide a description of the number of people receiving some but not all of the implementation strategies? OR  2: Does the practice provide a description of the strategy or strategies for some but not all of the groups? | 1^b^ |
|  |  | 1: Does the practice provide a description of the number of people receiving some or all of the implementation strategies? OR  2: Does the practice provide a description of the strategy or strategies for some or all of the groups? | 0^c^ |

^a^ : One condition present or no conditions present

^b^ One condition present

^c^: No conditions present

*Table 3. Overview of selected interventions in each GP practice*

|  | **Primary interven-tion** | **Description** | **Reported actions regarding implementation** | **Reported stakeholders in practice** |
| --- | --- | --- | --- | --- |
| A | SMS service | **Reminder,** which patients receive by SMS, two or three days before a diabetes consultation. The message includes the exact date and time of the consultation and the request to cancel the consultation if the patient is unable to attend | Regarding the accuracy of telephone numbers:  - Check availability of current telephone numbers  - Check correctness of current telephone numbers  - Registration in the appropriate field in the electronic medical record system  Regarding the delivery of SMS messages:  - Preparation of list for distribution  - Programming of individual messages for each separate patient, including scheduled date and time of consultation | Full practice team (medical assistants, nurse practitioners and general practitioners (GPs))  Medical assistants and nurse practitioners  Nurse practitioner  Nurse practitioner |
| B | Explora-tion of patient needs | This intervention consisted of several elements  **A. Small-scale patient panel:** focus group for in-depth exploration of patient needs regarding diabetes care  **B. Diabetes health market**  Large-scale patient meeting, based on input from patient focus group and approved by FP team: presentation of potential interventions, during which patients can express preferences for specific interventions  **C. Implementation of interventions most preferred by patients:**  -Diabetes educational training for patients, offered by diabetes federation  -Digital portal for patients (for further details see C) | - Selection and invitation of patients  - Reflection on generated output within GP team, decision-making regarding approval of potential interventions  - Selection and reservation of location  - Development of a meeting program  - Written invitation of all patients with type 2 diabetes and their primary caregivers  - Development of collaboration with local allied health, which includes several meetings  -Registration and referral of patients  - Personal training at practice location regarding use of digital portal  - Registration of patients in system  - Instruction of patients regarding use of system | GP  GP and colleague GPs within team  GP  GP  GP  GP  Nurse practitioner  Nurse practitioner  Nurse practitioner  Nurse practitioner |
| C | Type 2 diabetes e-portal | **Digital portal for patients** Functionalities include:  - Registration of health measures such as systolic blood pressure  - Registration of personal health targets  - Availability of educational videos | - Personal training at practice location regarding use of digital portal  - Registration of patients in system  - Instruction of patients regarding use of system | Nurse practitioner  Nurse practitioner  Nurse practitioner |
| D | Consulta-tion reduction | **Option** offered to patients during diabetes consultation, which includes reduction of consultation frequency from 4 to 1 or 2 annual consultations | - Identification and selection of patients who are eligible for intervention: stabilized T2DM and appropriate self-management skills  - Oral invitation during consultation | Nurse practitioners  Nurse practitioners |
